# A Systematic Treatise, Historical, Etiological, and Practical, on the Principal Diseases of the Interior Valley of North America, as They Appear in the Caucasian, African, Indian, and Esquimaux Varieties of Its Population

**Published:** 1850-10

**Authors:** 


### REVIEWS.

---

Art. IV.—*A Systematic Treatise, Historical, Etiological, and Practical, on the Principal Diseases of the Interior Valley of North America, as they appear in the Caucasian, African, Indian, and Esquimaux varieties of its Population.* By Daniel Drake, M. D. Cincinnati: Winthrop B. Smith & Co. Philadelphia: Grigg, Elliott & Co. New York: Mason & Law. 1850.

(continued.)

From the Balize, Dr. Drake ascends to the city of New Orleans, and gives a minute topographical description of this great Southern mart, in which he notices its position and plan, its lakes and swamps, its bayous, dikes, canals, street currents and inundations, its forests, the fevers of its swamp, its batture, its boats and shipping, the condition of the city, the composition of its society, its filth and fevers. The position of the city is a remarkable one. It is built upon land but recently reclaimed from the sea, and stands several feet below the river in its highest stages. Its latitude, the humidity of its climate, the nature of its soil, and its proximity to extended swamps, all point it out as the favorite seat of malarious diseases; and yet it is notorious that there are periods in which New Orleans is almost wholly exempt from such visitations. During the winter and spring its salubrity is probably much greater than that of more northern cities; but what is a more curious anomaly is, that its healthiest seasons are those which succeed inundations of the river. Dr. Drake remarks, that while the police of the city has been improved in latter years, the population has also greatly increased, so that along the river there has been a vast augmentation of foul and corrupting offals. It is along the latter, he adds, that yellow fever is most prevalent, as it is on the side of the city adjacent to the swamps that autumnal intermittents and remittents chiefly prevail. This is not the place to institute the inquiry whether the prevalence of the former in the neighborhood of the river is due to the local causes there abounding, or is attributable to its importation by ships arriving from more southern ports. This question is one which will come up and be discussed under the head of yellow fever.

From the following account of the angle of the Delta of the Mississippi one would hardly expect to learn that it was so exempt as it appears to be from miasmatic fevers:

"ANGLE OF THE DELTA.—This extends from the Bayou Plaquemine to the mouth of Red River, a distance of more than one hundred miles, and includes the important parishes of West Baton Rouge, Point Coupée, and the eastern part of Avoyelles, all in the State of Louisiana. The Mississippi, through nearly the whole length of this angle, flows close to its left bank, which is a continued tertiary bluff. The right bank is raised by a levee, which, however, does not afford full protection from overflows; for the bends in the river are here of the most remarkable kind; and consequently, from the retardation of the current, the levees are apt to give way; moreover, the banks of the Bayou Atchafalaya, which flows through the western part of the angle, are so low, that in the annual rise of the Mississippi they are deeply overflowed. Thus the settlements in this bottom are chiefly on the river bank, with a levee in front, and swamps, which generally dry up in autumn, and bayous, ponds, and lakes, in the rear. This portion of the Delta was settled by the French at an early period; and all the arable land has long been subjected to the action of the plow, and other agricultural implements, with full exposure to the rains and sun. Cotton, the former staple, has largely given place to sugar. The population of this region is entirely rural. There is not, I believe, a single town; but at the distance of fifty-five miles above Plaquemine, there is a public steamboat landing called Waterloo.

"From all that I have been able to learn, autumnal fever in this portion of the Delta is generally mild and not remarkably prevalent. Doctor Thomas Beaumont, who resided, when I saw him, on the tertiary plateau, several miles from the Delta, in the parish of East Baton Rouge, assured me, that malignant cases of autumnal fever were decidedly more frequent and fatal where he then lived, than in the Delta from which he had removed; and Doctor McKelvey, of St. Francisville, informed me on the authority of Doctor G. W. Smith, who had practiced his profession on the Point Coupée coast, and also on the opposite bluffs in West Feliciana, that the fevers of autumn were milder in the former than in the latter locality."

The medical topography of the great valley affords a number of facts which do not seem quite reconcilable with the commonly received theory of malaria. We take, as an instance, the Yazoo bottom, which is subject to wide annual inundations in the month of May, June, or July. Dr. Drake ascended the Yazoo river in the latter month, and found that the inundation extended, with but little dry land, to the Mississippi, distant thirty or forty miles to the west. "When the flood of the Mississippi subsides, the vast, temporary, and shallow lake is drained through the Yazoo; and before the month of September, much of the surface becomes so dry as to shrink until fissures are produced in the new deposits." All the circumstances most favorable to the production of malignant fevers are here present, and yet the testimony of the physicians of that region is, that the bottom is not more infected than the uplands. Yazoo city is built on a bluff above this bottom, in latitude 32° 40′, having drowned lands immediately opposite to it, in a western direction, across the river. It is thus exposed to the western, south-western, and north-western winds, all of which traverse this bottom. "Autumnal fever," says Dr. Drake, "prevails at Yazoo, but not beyond the degree of its prevalence on the uplands in the rear of the town." He adds that "although genuine and fatal cases of yellow fever have been introduced from the towns below, the disease has never spread."

No one will question that marshes, river-bottoms, stagnant water, and decaying vegetable matter, have an important agency in fostering disease; but facts such as these, which it would be easy to multiply to almost any extent, go to prove, we think, that we have still much to determine in regard to the laws of malaria. The "American Bottom," famous for its deep, rich soil, lies some degrees north of the Yazoo, but still within the zone of malignant fevers. It abounds in organic matters, and in sloughs, ponds, lakes, and bayous; and yet Dr. Drake did not learn that the French inhabitants, who settled in the bottom many years ago, "had been much affected with autumnal fever." The Americans who have more recently gone there "are sickly in autumn and summer." Dr. Farrer, of St. Louis, was able, in former years, to "distinguish, by their sallow complexion and languid aspect, the people of the American Bottom from those of the country back of the city." The French settlers, too, according to Peck, the historian of Illinois, many of them, "are rather dwarfish and stunted;" in all of which we see indubitable evidences of a subtile poison engendered by a peculiar soil, but which operates diversely in different localities. On the Yazoo bottom it excites a violent autumnal fever, and on the American bottom a mild intermittent, under the slow operation of which the body shrivels, and the face acquires a hue and expression which physicians recognise across the street.

Dr. Drake follows the Mississippi river to the highest settlement, and then recapitulates, in the following table, the relative prevalence of autumnal fever at the military posts which stand upon its banks:

| Posts. | N. Lat. | Distance from the Balize. | Elevation above the Gulf of Mexico. | Ratio per cent per annum of Intermittents. | Ratio per cent per annum of Remittents. |
|---|---|---|---|---|---|
| Fort Jackson, | 29° 29′ | 30 m. | 4 ft. | 114 | 15 |
| Baton Rouge, | 30° 36′ | 145 | 90 | 51 | 30 |
| Jefferson Barracks, | 38° 28′ | 1,378 | 460 | 34 | 16 |
| Fort Armstrong, | 41° 32′ | 1,722 | 550 | 17 | 10 |
| Fort Crawford, | 43° 03′ | 1,932 | 720 | 28 | 4 |
| Fort Snelling, | 44° 52′ | 2,192 | 850 | 4 | 2 |

From this table it will be seen, as already remarked, that there is a steady decrease of fever as we proceed north. Fort Crawford is unfavorably situated near the mouth of the Wisconsin, and affords an exception to the rule. Advancing north through fifteen degrees of latitude, and eight hundred and fifty feet of elevation, there is, with this exception, a regular decrease of intermittents from one hundred and fourteen per cent. down to four; and from the second post, Baton Rouge, a regular decrement of remittents from thirty per cent. to two.

This part of Dr. Drake's work affords many instructive illustrations of the bearing of mineral geology upon disease. Passing from the older rocks to the cretaceous formation in Alabama and Tennessee, it is found that fevers are both more common and more malignant. The towns which are included in this formation are Columbus, Georgia, on the left bank of the Chattahooche river; Montgomery, Wetumpka, Selma, Cahawba, Marion, Greensboro, Demopolis, Eutaw, Tuscaloosa, and Pickensville, in Alabama; Memphis, Somerville, Randolph, and Bolivar, in Tennessee; and Columbus, Yazoo, Pontotoc, and Holly Springs, in Mississippi. The whole of this region, according to Dr. Drake, who derived his knowledge from personal inquiry, "is infested with autumnal fever, beyond, perhaps, any other portion of the great valley." The following is his description of the country:

"The general surface of this cretaceous formation, near its northern margin, is hilly; further south, and in the neighborhood of the rivers, it is somewhat rugged, with extensive intervening plains, or table lands, from which the water, in many places, runs off with difficulty; and hence sluggish and swampy streams are not uncommon. As the strata are soft, the rivers, both small and large. have formed wide valleys, most of which are subject to inundation. Although the rocks, geologically speaking, belong to the cretaceous formation, no chalk has yet been discovered. The chief deposits are calcareous, and called by the people 'rotten limestone.' They consist of an argillaceous, friable carbonate of lime; but there are, likewise, soft sandstone, and strata of clay, sand, and gravel, of which some details may be given hereafter. Where the last two are at the surface, the rains percolate into the earth, and form permanent springs of pure, soft water; but where the rotten limestone shows itself at the surface, as it is free from fissures, and impervious to water, the springs are few, superficial, and transient."

The water of this region is not only of a bad taste, but seems to be positively noxious. Dr. Strudwich, of Demopolis, mentioned to Dr. Drake, that on a plantation of his, the operatives, who had been sickly under the use of the water of a "sipe-well," became healthy as soon as he had made an Artesian fountain.

The tertiary formation appears to be more healthy than the cretaceous, for the reason, perhaps, that more of its surface consists of sand, and being dry and sterile is wanting in the elements of malarious disease. But where the country is fertile, fevers of the worst type prevail. Probably no river in the great valley is more sickly than the Alabama, which runs through the cretaceous and tertiary formations.

Pascagoula river runs in all its course through tertiary formations. Its mouth is in latitude 30° 30′, and its extreme sources are in 32° 40′ north. Speaking of that part of the country on the Pascagoula which lies near the Gulf, Mr. Darby says: "its unfruitfulness is counterbalanced to the inhabitants by the health they enjoy." According to the testimony of all, the basin of this river is but little affected with the fevers of autumn, and in its salubrity, affords, as remarked by our author, "instructive evidence of the connection, in the manner of cause and effect, between the topographical condition of a southern region and its bilious fevers."

Dr. Drake concludes his account of this part of the valley with the following general statements:

"1. The whole region which has been surveyed, lies south of the thirty-seventh degree of north latitude, and its broadest part is in the thirty-second; it is, therefore, a southern region.

"2. Its elevation above the Gulf of Mexico is but small, not averaging more than four hundred feet.

"3. It has an inclination to the south; which is true even of those parts which discharge their waters westerly and south-westerly into the Mississippi.

"4. It is composed of the (geologically) recent cretaceous and tertiary formations, which are friable in texture and miscellaneous in composition, still containing remains of organic matter.

"5. As a consequence of this structure, its streams have wide alluvions, and sluggish currents, which lead to frequent valley-inundations.

"6. It is undeniable, that this great region is more generally and seriously infested with autumnal fever, than any other portion of the interior valley of North America.

"7. Most of it has been settled within the last thirty years, and new plantations are still forming.

"It is, therefore, what we, provincially, call a new country, and with the progress of cultivation, may become much healthier. Most of its streams, however, will long continue to overflow their low, broad, alluvial bottoms, and thus thread the higher lands with lines of pools and swamp, which, under the influence of a southern sun, will of necessity send forth the efficient cause of autumnal fever. The question, moreover, may be raised, whether, in summer and autumn, there may not be telluric emanations, from the unconsolidated strata of the comparatively recent tertiary and cretaceous formations, which the older carboniferous, Devonian, and Silurian formations do not send forth. I would not venture to answer this question affirmatively; but if such be the case, the region we have surveyed is permanently exposed to an additional cause of insalubrity."

The next section reviewed is the Ohio basin, which he thus contrasts with the preceding:

"The Ohio basin differs, in many respects, from the more southern basins over which we have traveled. Its general elevation above the level of the sea, excluding its mountains, is more than twice as great as the regions we have just left—that is, from seven hundred to one thousand feet; while the mountain borders to the east and south-east, rise from two thousand five hundred to five thousand feet. The north-west portions, in Illinois, Indiana, and Ohio, embrace tracts of level land, not unlike the plains of Alabama; but south of the Ohio river, the surface is everywhere ridgy, rising eastwardly into the mountainous; a character which belongs equally to the eastern portions of the region north of the Ohio.

"Geologically, the difference is equally great. A very small part, near the mouths of the Ohio and Tennessee rivers, presents the cretaceous formation, on which we have dwelt so long; all the rest offers at the surface older geological formations. The western, southern, and eastern parts embrace extensive coal deposits, with their accompanying sandstones, shales, and limestone; the central portions show at the surface Devonian sandstones and shales, of an older geological date; and large tracts of Silurian limestone, still older in the geological series, are found at the surface; the carboniferous and Devonian formations seeming to have been washed away. The oldest of these Silurian or transition rocks bulge up in, and a little south of, what has been designated as the geographical center of the basin. The rocks of these various formations are firmly indurated, compared with the cretaceous and tertiary deposits, and have, therefore, been acted upon much less in a lateral direction. Hence most of the valleys are mere ravines, compared with those which have been excavated through loose and friable strata. This is especially true of that part of the basin which lies south of the Ohio river. To its north, however, in Illinois, Indiana, and Ohio, there are deep and extensive deposits of drift or diluvium, in which the streams have excavated wide valleys, and formed alluvial bottoms of corresponding breadth. All parts afford springs, though not of equal copiousness and permanence, and well-water can everywhere be obtained. The creeks and rivers rise rapidly; and the range between low and high water is great. In the northern margin of the basin, there are many small lakes, or, more properly speaking, large ponds, and numerous swamps of still greater area; but in the basin generally they are not found."

But we must quit topography, and proceed to notice the climate of the valley.

Climate, in physical geography, expresses a zone of the earth "parallel to the equator, and of such a breadth that the longest day in the parallel nearest the pole is half an hour longer than that nearest the equator;" but in etiology the term simply expresses states of the atmosphere. These, as enumerated by Dr. Drake, "consist in varying quantities or qualities of certain elements of the air itself—its caloric, light, and electricity; its aqueous vapor, fogs, mists, and clouds; its dews, rain, hail, frost, and snow; its weight and density; its movements or winds; its factitious gases, and mechanical impurities; all of which," as he correctly remarks, "may be very different in different times or places of the same geographical climate, and nearly the same in different zones."

We have alluded to the length of that part of this volume which treats of the climate of the great interior valley. We have no doubt that the subject is discussed at greater length than many readers would have chosen; but there is this to be said of it, that the laws of climate over a field so vast as this valley could not be developed in a few pages. It has been the aim of the author to ascertain and unfold these laws throughout the whole extent of this wide region, and the interesting result is brought out, that almost every physician, knowing his latitude, longitude, and elevation, with this volume in his hand may estimate, without himself making observations, the character of the climate in which he lives. It will be admitted that such a result was worth a good deal of toil, and that a book which contains such a formula merits the favor of every scientific man.

The temperature is shown by meteorological tables kept at various posts, from north latitude 10° to 74°. According to these tables, the ratio of decrease of mean temperature is not uniform as we go north from the equator. Assuming with Humboldt, that 82° is the mean heat of equatorial regions up to 10° north, the decrease from this latitude to Melville Island, the mean annual heat of which is more than one degree below zero, ought to be 1° .28 for each degree of latitude; but there are many exceptions to the law. In some places, the decrement is only thirty-three hundredths of a degree of temperature for every degree of latitude; while in others it is 2° .04, 2° .10, 2° .17, 2° .28, and 2° .67. But taking all the data, Dr. Drake has been able to construct a theoretical table of mean temperatures which corresponds very closely with the tables made out from observation. In making the calculation, he employs three decrements of temperature; the first, for the equatorial belt of thirty-three hundredths of a degree for every degree of latitude; the second of 1° .16 (one degree sixteen hundredths) for the region extending from the equatorial belt to New Orleans; and the third of 1° .655 (one degree six hundred and fifty-five thousandths) for the region lying between 30° and 48° north.

In reference to the value of his calculations Dr. Drake remarks:

"Although these tables present only approximations to the truth, they will not be found useless to those who live in places where no thermometrical observations have been made. Should the reader wish by their aid to determine (approximately) the mean temperature of any particular spot, he must know its latitude, and, if it be beyond the forty-eighth parallel, its longitude, and in all cases its probable elevation above the level of the sea; when, by a resort to the ratios of the table, he can readily make the calculation. In the first section (within the tropics) one minute of latitude diminishes the temperature 0° .0055 (fifty-five ten thousandths of a degree,) which being multiplied by the number of minutes which his station lies above any given latitude of the table, will show him what deduction to make from the temperature of that latitude. For example, the temperature of the twentieth parallel, is 78° .82; if it be required to know the temperature of N. Lat. 20° 45′, the ratio for one minute, .0055, must be multiplied by 45′, which gives .25, which being substracted from 78° .82, leaves 78° .57 as the amount sought.

"In the second section of the table, the ratio of 1° .16 for a degree of latitude, gives 0° .193 (one hundred and ninety-three ten thousandths of a degree) for every minute; which must be, as in the other case, multiplied by the number of minutes of any particular station above a given parallel, and the product subtracted from the temperature of that parallel.

"In the third section of the table a degree of latitude reduces the mean heat 1° .655, which is, for each minute, 0° .0276 (two hundred and seventy-six ten thousandths,) to be multiplied, as in the other cases, by the number of minutes from the nearest parallel below, and the amount subtracted from the heat of that parallel.

"But in all cases allowance must be made for the difference of altitude. In the first two sections of the table, the ratios refer to the level of the sea; in the third, to an average elevation of six hundred feet. Let us take an example. Pompey, in N. Lat. 42° 56′, has, from observation, a mean temperature of 42° .84, which is 6° .03 less than that from calculation; but the altitude of Pompey, one thousand three hundred feet, or seven hundred feet above the average, reduces its temperature 3° .50, and this being added to the temperature from observation, raises it 46° .34." *(To be concluded.)*

